# Abdominal muscle fatigue following exercise in chronic obstructive pulmonary disease

**DOI:** 10.1186/1465-9921-11-15

**Published:** 2010-02-04

**Authors:** Nicholas S Hopkinson, Mark J Dayer, John Moxham, Michael I Polkey

**Affiliations:** 1 National Heart and Lung Institute, Imperial College, Royal Brompton Hospital, Fulham Rd, London SW3 6NP, UK; 2 King's College Hospital, Denmark Hill, London SE5 9RS, UK

## Abstract

**Background:**

In patients with chronic obstructive pulmonary disease, a restriction on maximum ventilatory capacity contributes to exercise limitation. It has been demonstrated that the diaphragm in COPD is relatively protected from fatigue during exercise. Because of expiratory flow limitation the abdominal muscles are activated early during exercise in COPD. This adds significantly to the work of breathing and may therefore contribute to exercise limitation. In healthy subjects, prior expiratory muscle fatigue has been shown itself to contribute to the development of quadriceps fatigue. It is not known whether fatigue of the abdominal muscles occurs during exercise in COPD.

**Methods:**

Twitch gastric pressure (TwT10Pga), elicited by magnetic stimulation over the 10^th ^thoracic vertebra and twitch transdiaphragmatic pressure (TwPdi), elicited by bilateral anterolateral magnetic phrenic nerve stimulation were measured before and after symptom-limited, incremental cycle ergometry in patients with COPD.

**Results:**

Twenty-three COPD patients, with a mean (SD) FEV_1 _40.8(23.1)% predicted, achieved a mean peak workload of 53.5(15.9) W. Following exercise, TwT_10_Pga fell from 51.3(27.1) cmH_2_O to 47.4(25.2) cmH_2_O (p = 0.011). TwPdi did not change significantly; pre 17.0(6.4) cmH_2_O post 17.5(5.9) cmH_2_O (p = 0.7). Fatiguers, defined as having a fall TwT10Pga ≥ 10% had significantly worse lung gas transfer, but did not differ in other exercise parameters.

**Conclusions:**

In patients with COPD, abdominal muscle but not diaphragm fatigue develops following symptom limited incremental cycle ergometry. Further work is needed to establish whether abdominal muscle fatigue is relevant to exercise limitation in COPD, perhaps indirectly through an effect on quadriceps fatigability.

## Background

Chronic obstructive pulmonary disease (COPD) is characterized by damage to airways and lung parenchyma, which leads to expiratory flow limitation. As expiratory flow is volume-dependent, increased ventilatory demands are met by an increase in operating lung volumes. This dynamic hyperinflation places both elastic and resistive loads on the respiratory muscles and increases the disparity between neural drive and mechanical output [1]. There has been long-standing interest in the role of the respiratory muscles in contributing to ventilatory limitation and task failure, both in health and disease [2,3].

Peripheral muscle fatigue is defined as a reversible loss of the ability to generate force, resulting from activity under load [2]. The diaphragm is the principal inspiratory muscle and it is possible to induce diaphragm fatigue in healthy subjects through breathing against an inspiratory load or by maximum voluntary ventilation [4,5]. Diaphragm fatigue also occurs at high levels of whole body exercise [6-8]. However in COPD, it has been shown that despite the diaphragm being loaded during exercise [9], low frequency fatigue, demonstrated by a persistent fall in response to supramaximal nerve stimulation, does not occur following either treadmill or cycle exercise [10,11]. Likewise, maximum voluntary ventilation did not induce diaphragm fatigue in a study of six patients with severe COPD [12]. Moreover, even in patients with COPD who fail a trial of weaning from mechanical ventilation, low frequency diaphragm fatigue was not observed [13]. Taken together, these data suggest that diaphragm fatigue is unlikely to be relevant to exercise limitation in COPD, perhaps because of a protective effect of hyperinflation and consequent muscle shortening [14].

During quiet breathing in healthy subjects, expiration is passive, driven largely by lung elastic recoil, but as ventilation increases the abdominal muscles are recruited to increase expiratory flow rate [15]. Few data exist regarding the role of expiratory muscle fatigue, but it has been demonstrated that maximum voluntary ventilation loads the abdominal muscles, slowing their relaxation rate and causing low frequency fatigue to develop [5,16,17]. High intensity, whole-body exercise also causes expiratory muscle fatigue to develop in healthy individuals [18,19]. In COPD, the abdominal muscles are frequently recruited even during resting breathing [20]. When walking to exhaustion, inspiratory work of breathing in COPD rises rapidly but then plateaus, whereas expiratory muscle recruitment and pressure time product continue to rise [21] and in some patients slowing of the expiratory muscle maximum relaxation rate has been noted [22].

Low frequency fatigue (LFF) describes the loss of force generated in response to low stimulation frequencies (10-20 Hz), which are the typical motor neuron firing frequencies during human skeletal muscle activity. LFF in a muscle can be identified by measuring the reduction in the force elicited by a single stimulus applied to a peripheral nerve supplying that muscle, before and after exercise (or other contractile activity), provided the same stimulus is given before and afterwards. A convenient way to do this is to give a stimulus which activates all nerve and muscle fibres (a supramaximal stimulus)[23]. For skeletal muscle LFF is typically assessed 20 minutes after exercise to allow the effects of exercise induced potentiation to wear off [24].

This study was intended to investigate whether the increased loading that the expiratory muscles are subject to during exercise in COPD, would lead to the development of abdominal muscle fatigue, assessed using the technique of magnetic stimulation of the lower thoracic nerve roots.

## Methods

Patients with COPD, defined according to GOLD criteria[25] were recruited from outpatient clinics. Patients were excluded if they had had symptoms suggestive of an acute exacerbation in the previous month. The Research Ethics Committee of The Royal Brompton Hospital approved the study. All patients gave written informed consent. Some of the baseline data from these subjects has been reported previously [26].

Spirometry, plethysmographic lung volumes and gas transfer (Compact Lab System, Jaeger, Germany) as well as arterialized capillary blood gas tensions were measured as described previously [26]. Fat free mass (FFM) was determined using bioelectrical impedance analysis (Bodystat 1500, Bodystat, Isle of Man, UK) and a disease specific regression equation [27].

Following the placement of oesophageal and gastric balloon catheters[28] maximum inspiratory (PImax), expiratory (PEmax) [29], sniff nasal (SNiP), transdiaphragmatic (SnPdi)[30] and cough gastric (CoughPga)[31] pressures were determined. Pressure signals were amplified and passed to a computer running LabView 4.1 software (National Instruments, Austin, Texas, USA),

After performing the volitional tests, subjects remained seated quietly for twenty minutes to depotentiate their respiratory muscles. Diaphragm strength was assessed as the unpotentiated response elicited by bilateral, anterolateral, magnetic phrenic nerve stimulation (TwPdi) at resting end expiration, using a pair of 45mm figure of eight coils each powered by a Magstim 200 monopulse unit (Magstim Ltd, Whitland, UK) delivering an output 100% of maximum with patients seated upright in a straight-backed chair [32].

Abdominal muscle strength was assessed using the gastric pressure response to stimulation, delivered to the nerve roots supplying the abdominal muscles, at the level of the 10^th ^thoracic vertebra (TwT_10_Pga) by a circular coil. Coil position was adjusted to produce the maximal response in gastric pressure. Stimulations were performed at total lung capacity, with the patient seated upright astride the chair. Subjects were instructed to inhale to total lung capacity fully and then relax with a closed glottis. Care was taken to ensure that the subject maintained the same posture and coil position was marked with indelible pen.

The exercise protocol used has been described elsewhere [26]. Briefly, it involved an initial two minute rest period followed by unloaded cycling for 30 seconds and then increments of 5 W every 30 seconds subsequently. A mouthpiece connected to an Oxycon device (Jaeger, Germany) was used for breath-by-breath metabolic measurements of oxygen consumption (VO_2_) and CO_2 _production (VCO_2_). Subjects performed an inspiratory capacity manoeuvre every minute to assess dynamic hyperinflation. EELV was calculated by subtracting inspiratory capacity from total lung capacity (as the latter does not change during exercise[33,34]). The reason given for stopping was documented.

Following exercise, subjects sat quietly for 20 minutes to depotentiate before the magnetic stimulations were repeated. On both occasions, the phrenic nerve stimulations were performed before the thoracic nerve root stimulations.

### Statistical analysis

Values before and after exercise were compared using paired t tests. Correlations between percent change in TwT_10_Pga and both baseline parameters and exercise parameters were sought using linear regression analysis. Individuals where the TwT_10_Pga fell by >10% were defined as 'fatiguers' and compared to 'non-fatiguers' using an appropriate test for paired comparison. Values are expressed as mean (SD) and a p value of < 0.05 was taken to be significant.

## Results

Twenty-three COPD patients (17 male) with a mean(SD) FEV_1 _40.8(23.1)% took part in the study. Baseline characteristics and exercise performance are given in Table 1. 10 patients reported that they stopped because of breathlessness, 5 because of leg fatigue and 8 because of a combination of the two. During exercise, significant dynamic hyperinflation occurred, with end expiratory lung volume (EELV) rising from 5.97(1.65) litres to 6.62(1.95) litres (p < 0.0001).

**Table 1:** Participant characteristics and exercise parameters:

	Mean(sd) n = 23	Non-fatiguers n = 15	Fatiguers n = 8	P
Age (years)	61.8 (25.2)	63.1 (8.5)	59.4(12.6)	0.4

Pack years smoked	50.5(23.3)	54.1(25.6)	43.7(17.6)	0.3

BMI (kg.m^-2^)	23.3 (5.0)	24.1(3.5)	21.7(7.1)	0.3

FFMI (kg.m^-2^)	16.1(2.0)	16.3 (1.6)	15.5 (2.8)	0.4

*Lung function*				

FEV_1 _%predicted	40.8(23.1)	45.8 (25.2)	31.6 (15.9)	0.17

FVC %predicted	85.1(29.3)	93.1 924.8)	70.1 (11.6)	0.07

TLC %predicted	131.4(17.5)	130.3 (16.5)	133.3 (20.4)	0.7

RV/TLC	58.2(9.2)	57.5 (8.0)	59.6 (11.7)	0.6

FRC %predicted	182.0(41.2)	177.9 (35.5)	189.6 (52.4)	0.5

TLco_c _%predicted	39.6(16.4)	45.4 (16.3)	30.0 (10.9)	0.03*

Kco_c _%predicted	45.9(18.7)	51.8 (19.0)	34.9 (12.9)	0.04*

PaCO_2 _(kPa)	4.9(0.6)	4.7 (0.6)	5.2 (0.4)	0.06

PaO_2 _(kPa)	9.9(1.1)	9.8 (1.1)	10.0 (1.2)	0.7

*Muscle strength*				

PImax (cmH_2_O)	53.7(21.2)	50.7 (18.0)	58.5(26.1)	0.4

PEmax (cmH_2_O)	80.9(26.3)	77.5(27.4)	86.4(25.2)	0.5

SnPdi (cmH_2_O)	94.2(16.1)	92.7(12.3)	97.0(22.5)	0.6

Cough Pgas (cmH_2_O)	241.0(60.3)	242.7(66.3)	237.6(49.5)	0.9

TwT10Pgas (cmH_2_O)	51.3 (27.1)	47.7 (24.5)	58.1(31.8)	0.4

TwPdi (cmH_2_O)	17.0 (6.4)	17.4 (6.2)	16.2 (6.9)	0.7

QMVC (kg)	32.3 (9.6)	32.1 (10.0)	32.6 (9.5)	0.9

*Exercise parameters*				

Peak VO_2 _(ml.kg^-1^/min)	11.5 (3.3)	12.1 (3.3)	10.4 (3.0)	0.2

Peak VCO_2 _(ml.kg^-1^/min)	11.1 (3.9)	11.6 (4.2)	10.1 (3.2)	0.4

Peak workload (W)	53.5(15.9)	57.0 (16.7)	47.0 (13.0)	0.16

Peak VE (l/min)	29.9 (9.2)	32.3 (9.4)	25.5 (7.6)	0.09

Rest EELV (l)	5.97(1.65)	5.84 (1.5)	6.22 (2.0)	0.6

Peak EELV (l)	6.62(1.95)	6.46 (1.9)	6.91 (1.1)	0.6

Δ EELV (%)	10.4 (8.2)	9.7 (6.1)	11.6 (11.6)	0.7

Δ TwPdi (%)	2.2. (15.9)	+7.7 (14.9)	-7.9 (13.2)	0.02*

Δ TwT10Pgas (%)	-7.2 (15.6)	0.3 (10.8)	-21.3 (13.5)	<0.001*

BMI body mass index, FFMI fat free mass index, FEV_1 _forced expiratory volume in one second, FVC forced vital capacity, TLC total lung capacity, RV residual volume, FRC functional residual capacity, TLco_c _carbon monoxide transfer factor corrected for haemoglobin, Kco_c _carbon monoxide transfer coefficient corrected for haemoglobin, PaO_2 _partial pressure of oxygen, PaCO_2 _partial pressure of carbon dioxide, PImax maximum inspiratory pressure, PEmax maximum expiratory pressure, SnPdi sniff transdiaphragmatic pressure, Pgas gastric pressure, TwPdi twitch transdiaphragmatic pressure, QMVC quadriceps maximum voluntary contraction force, VO_2 _oxygen consumption, VCO_2 _carbon dioxide production, EELV end expiratory lung volume.

**Figure 1:**
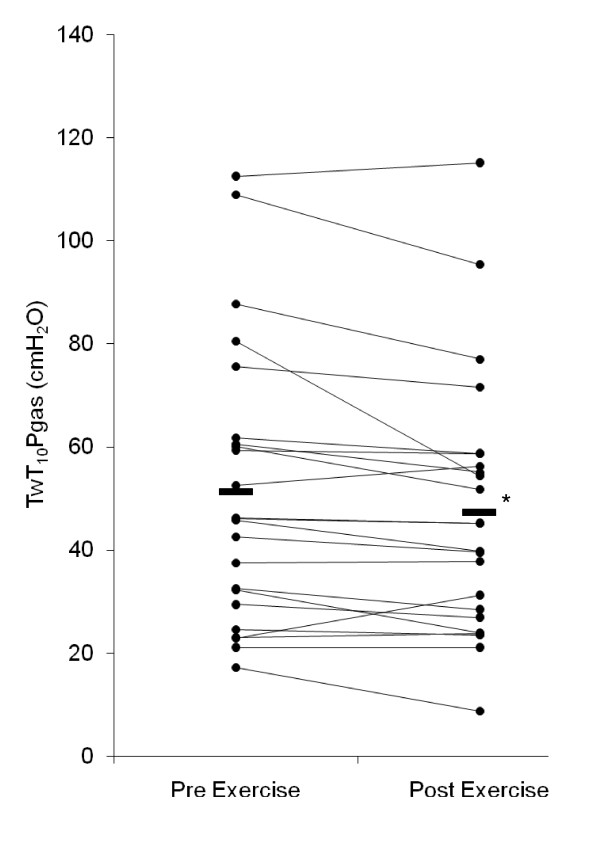
**Twitch gastric pressure before and after exercise**. Twitch T10 gastric pressure fell significantly following symptom limited cycle ergometry in 23 patients with COPD. (*p = 0.011).

Following exercise, TwT_10_Pga fell from 51.3(27.1) cmH_2_O to 47.4(25.2) cmH_2_O (p = 0.011) (Figure 1). In 8 patients it fell by more than 10% from baseline. The gastric pressure at which T10 stimulations were administered did not differ significantly; pre 22.3(6.6) cmH_2_O vs post 22.4(7.3) cmH_2_O.

There was a weak inverse correlation between the percent fall in TwT_10_Pga and Kco_c _(r^2 ^0.19 p = 0.04) but change in TwT_10_Pga did not correlate with any other baseline parameter. There was also no correlation with parameters measured during exercise including , , VE, exercise duration, the degree of dynamic hyperinflation that occurred or the reason for stopping exercise.

Subjects with at least a 10% fall in TwT_10_Pga 'fatiguers' were compared with 'non-fatiguers' (Table 1). Gas transfer was significantly lower in the fatiguing group and lung volumes tended to be worse, though the latter differences were not statistically significant. There was no difference between the two groups in terms of exercise parameters, in dynamic lung volume changes or reasons given for stopping.

There was no significant change in TwPdi following exercise - pre 17.0(6.4) cmH_2_O post 17.5(5.9) cmH_2_O (p = 0.7).

Portable equipment needed to perform the invasive measurements *during *exercise in the exercise lab was not available when some of the subjects were studied. Oesophageal and gastric pressure measurements during exercise were therefore available in 14 subjects, 6 of whom were fatiguers. In this subgroup, gastric pressure time product increased from 134 (160) cmH_2_O.sec.min^-1 ^at rest to 555(332) cmH_2_O.sec.min^-1 ^during the last 30 seconds of exercise (p < 0.0001). Neither absolute PTPga, nor change in PTPga, nor the amplitude of the gastric pressure swing with expiration was associated with change in TwT_10_Pga.

## Discussion

We found that in patients with COPD, twitch gastric pressure fell following symptom-limited cycle ergometry, whereas twitch transdiaphragmatic pressure did not, indicating that low frequency fatigue had developed in the abdominal muscles but not the diaphragm. The mean change was small and was not associated with any parameter measured during exercise. Abdominal muscle fatigue was more likely to occur in patients with the lowest gas transfer.

### Significance of findings

Our results suggest that fatigue of the abdominal muscles, the main muscles of expiration, can develop in COPD patients exercising to exhaustion on a cycle ergometer. Although not measured during exercise, the severity of our patients' COPD, judged by FEV_1_, and the shape of their flow volume curves makes the likelihood of their having flow limitation extremely high. Accepting this assumption, the presence of expiratory flow limitation means that increased abdominal muscle recruitment during exercise would not increase expiratory flow rates and as such the activation may to some extent be 'futile', which is not the case in normal subjects, in whom the distinction into inspiratory and expiratory is not absolute. During exercise, at least in normal subjects, the expiratory muscles act as accessory muscles of inspiration by reducing end expiratory lung volume, so that the diaphragm is lengthened to an optimum position. Thus their relaxation assists the diaphragm during inspiration, possibly allowing high levels of ventilation to be sustained for a longer period [35].

Abdominal muscle fatigue could be relevant to exercise performance in COPD either because it limits ventilation directly, or because of indirect effects. There is evidence in healthy subjects that high intensity exercise produces expiratory muscle fatigue [18,19] and that fatigue of the expiratory muscles can influence exercise performance [36,37]. Moreover in patients with a congenital weakness of abdominal muscles, the prune belly syndrome [38], peak exercise performance is reduced. Suzuki *et al *found that fatiguing the abdominal muscles with sit ups to task failure, caused a reduction in both PEmax and TwT_10_Pga, but did not reduce subsequent performance of MVV, which argues against a direct effect on ventilatory capacity as a mechanism of exercise limitation [39].

Fatigue of the expiratory muscles has been shown to increase sympathetic vasoconstrictor outflow to peripheral muscles [40], which could promote limb muscle fatigue. Consistent with this, a greater degree of quadriceps fatigue occurred after exercise in subjects cycling having first undergone an expiratory muscle fatiguing protocol, than following an equivalent exercise duration when not first fatigued [36]. Interestingly, in that study subjects exercising with prior expiratory muscle fatigue experienced both more dyspnoea and greater leg discomfort. In the present study quadriceps fatigue was not measured, so we cannot comment on any possible relationship between abdominal and limb muscle fatigue in COPD though this would clearly be an interesting area for future work.

The fall in TwT_10_Pga was smaller than that observed following exhaustive exercise in healthy subjects exercising to exhaustion [18,19]. This may be because of differences in the exercise protocol (incremental vs. endurance) or in the symptoms limiting exercise.

The observation that 'fatiguers' had worse lung function parameters is interesting. This was not reflected in differences in the symptoms limiting exercise, the degree of dynamic hyperinflation that occurred or in oxygenation during exercise. Gas transfer has been associated with impairment of fat free mass [41] in COPD but neither this nor quadriceps strength differed between the two groups. We also note that this group had a mean 7.9% fall in TwPdi, while the non-fatiguers had a mean 7.7% increase (making a mean difference of 15.6% compared with 21.6% for TwPga). This relationship was not significant when the two parameters were considered as continuous variables so should be treated with caution. It does raise the possibility that a sub-population of patients with COPD might be particularly sensitive to developing respiratory muscle fatigue during exercise, perhaps because the demands of the contracting quadriceps exert a 'steal' phenomenon from both inspiratory and expiratory muscle groups.

Our findings are consistent with previous work showing that the diaphragm does not fatigue following exercise in COPD [10]. This may be because of muscle adaptations including an increased proportion of type I fatigue resistant muscle fibres, or because muscle shortening due to lung hyperinflation protects against fatigue [42]. Conversely abdominal muscles lengthen during hyperinflation potentially rendering them more susceptible to fatigue, though we saw no relationship between dynamic hyperinflation and the propensity to abdominal muscle fatigue. We are not aware of any data regarding the fibre type of abdominal muscles in COPD (in health the fibre distribution is similar to the quadriceps [43]), but their strength is preserved in the condition as evidenced by normal cough gastric pressures [31].

### Methodological issues

A key task was to ensure that the conformation of the abdomen was similar before and after exercise. Care was taken to ensure that the stimuli were delivered in the same way, with the coil and patients in the same position. We did not repeat measurements of lung volumes following exercise, but it is known that total lung capacity does not change significantly either during or after exercise in patients with COPD [34,44]. The observation that the gastric pressure at which stimulations were delivered was the same pre- and post-exercise also suggests that the conformation of the abdominal compartment was similar in both conditions. This was also the case for end-expiratory oesophageal pressure when phrenic nerve stimulations were delivered. The absence of change in TwPdi or TwPoes also argues against significant lung volume change at the point of measurement, since these variables are known to be sensitive to lung volume change [45].

For reasons of tolerability we did not formally assess the supramaximality of the magnetic stimulation in either the phrenic or thoracic nerve root stimulation. In the case of magnetic phrenic nerve stimulation this has been demonstrated in numerous previous studies [32,46-50]. For thoracic nerve root stimulation, a plateau in M-wave response has been observed by a number of authors [18,19,36] with no change in M-wave occurring after exercise [16,18,19,36], suggesting that any exercise induced fall in TwT_10_Pga is due to a reduction in contractility rather than a reduction in electrical transmission.

It is also possible that the extent of fatigue was 'underestimated' because of the use of unpotentiated twitches, as the change in the (larger) potentiated twitches following fatiguing tasks tends to be more pronounced [51,52]. However we also note that the recent vogue for using potentiated twitches [51] is predated by the original description of magnetic stimulation techniques in which unpotentiated twitches were universally used (for example[10,12,23]), precisely because investigators wished to be confident that true fatigue (rather than a modulating effect of potentiation) had occurred.

Finally, we chose to deliver TwT10 stimulations at TLC rather than FRC in this study, because in pilot work the response was larger, and also because TLC is considered to be a fixed volume in COPD unlike FRC which is known to vary with minute ventilation. We think variance from this source is likely to have been modest, both because the length-tension relationship for the abdominal muscles is considerably less important than for the diaphragm [53] and because our COPD patients, by virtue of resting hyperinflation (Table 1) had an FRC which was markedly closer to TLC than would be observed in healthy subjects. Other studies have used stimulation at FRC, which precludes direct comparison of the amplitude of the twitches [16-18]. Although we did not study repeatability in this population, the reproducibility of response to TwT10 stimulation has been confirmed in healthy subjects [16,18,36].

## Conclusions

Expiratory muscle fatigue occurs in patients with COPD exercising to exhaustion, but it does not necessarily follow that this fatigue is relevant to exercise performance in COPD. If this were to be the case, it may well be through increasing quadriceps fatigability through enhanced sympathetic activation, rather than via a direct effect on ventilatory capacity. Further studies are needed to establish whether expiratory muscle fatigue has an impact on quadriceps fatigability in this population.

## Competing interests

The authors declare that they have no competing interests.

## Authors' contributions

NSH, MD, JM and MIP conceived the study; NSH and MD performed the study measurements and the data analysis. NSH wrote the first draft of the paper to which all authors subsequently made contributions. All authors read and approved the final manuscript.
